# Evaluation of biochars from different stock materials as carriers of bacterial strain for remediation of heavy metal-contaminated soil

**DOI:** 10.1038/s41598-017-12503-3

**Published:** 2017-09-21

**Authors:** Ting Wang, Hongwen Sun, Xinhao Ren, Bing Li, Hongjun Mao

**Affiliations:** 1 Key Laboratory of Pollution Processes and Environmental Criteria of Ministry of Education of the People’s Republic of China, College of Environmental Science and Engineering, Nankai University, Tianjin, 300071 People’s Republic of China; 2 Centre for Urban Transport Emission Research, College of Environmental Science and Engineering, Nankai University, Tianjin, 300071 People’s Republic of China; 3 School of Environmental Science and Engineering, Shaanxi University of Science and Technology, Xi’an, 710021 People’s Republic of China

## Abstract

Two kinds of biochars, one derived from corn straw and one from pig manure, were studied as carriers of a mutant genotype from *Bacillus subtili*s (B38) for heavy metal contaminated soil remediation. After amendment with biochar, the heavy metal bioavailability decreased. Moreover, the heavy metal immobilization ability of the biochar was enhanced by combining it with B38. The simultaneous application of B38 and pig manure-derived biochar exhibited a superior effect on the promotion of plant growth and the immobilization of heavy metals in soil. The plant biomass increased by 37.9% and heavy metal concentrations in the edible part of lettuce decreased by 69.9–96.1%. The polymerase chain reaction-denaturing gradient gel electrophoresis (PCR-DGGE) profiles revealed that pig manure-derived biochar could enhance the proliferation of both exotic B38 and native microbes. These results suggest that B38 carried by pig manure-derived biochar may be a promising candidate for the remediation of soils contaminated by multiple heavy metals.

## Introduction

Currently, heavy metal pollution in farmland has become a serious environmental issue in China and other developing countries^1^. The immobilization of heavy metal contaminations is recognized as a practicable technology because the agricultural activity does not need to be interrupted during the remediation period. In addition to the well-known chemical sorbents, some types of microorganisms have been found to have a high affinity for heavy metals and can biosorb or precipitate heavy metals by various mechanisms^2^. The bioaugmentation of microorganisms is environmentally friendly and is therefore an ideal option for lowering the hazardous effects of heavy metals on living beings without deteriorating soil properties^3^.

A problem encountered in bioaugmentation is the toxicity of heavy metals, which causes high pressure on the growth of bioaugmented microbes. Therefore, a crucial step is the acquirement of high heavy metal-tolerant microbial species. The main breeding methods include acclimatization, mutagenesis and genetic engineering^4^. However, the acclimatization of strains is time-consuming and inefficient, and genetic engineering is complex and still in its early stages of development. Thus, mutation breeding is a promising option for the enhancement of microbial activity. Several recombination technologies of increasing the induced mutation frequency have been developed, such as X-ray irradiation, ultraviolet (UV) irradiation, ethyl methane sulfonate (EMS) treatment and HNO_2_ induction^5,6^. UV-irradiation induced mutation is the simplest and most effective physical mutation method, and it has been widely used in the breeding of microbes for industrial use^7–9^. However, this technology has seldom been used in mutation for improving the heavy metal resistibility of microorganisms^10,11^.

Another limitation of bioaugmentation is that the contaminated soils are usually nutrient deficient and cannot support the rapid growth of the bioaugmented microbes^12^. A strategy to stimulate the metabolism and proliferation of microorganisms is the addition of nutrients, i.e., biostimulation^13^. Several matrixes have been tested, including pure nutrients, such as glucose, and wastes from agriculture^14^ and industry^15^. The combined technology of bioaugmentation assisted by biostimulation is a high-efficiency integrated technology and implies a promising approach to the bioremediation of heavy metal-contaminated soils.

Biochar is a carbonaceous material produced from oxygen-limited pyrolysis of biomass under low temperatures^16^. The relative carbonized and non-carbonized fractions determine its sorption behavior^17^. The organic carbon components of biochar can stabilize heavy metals by electrostatic interactions, ionic exchange, sorptive interaction, and the specific binding of metal ions by surface ligands^18,19^. Biochar contains large amount of surface adsorption sites, such as carboxyl and hydroxyl groups, which contain many active oxygen atoms^20^. The surface functional groups in biochar could control the levels of heavy metal content by forming specific metal-ligand complexes in the soil; during continuous operation, these mechanisms could cause mineral precipitation on the soil particle surface through a monolayer adsorption process^21^. Generally, a lower pyrolysis temperature is favorable for stabilizing the heavy metals and releasing P, K, Ca and other plant nutrients to soil^21^. Such a capability may also depend on the original feedstock of the biochar^22,23^. Biochars derived from plant residues commonly contain low amounts of inorganic moiety (ash) and high cation exchange capacity (CEC)^24,25^. Biochars derived from livestock manure have quite different compositions compared to those from plant residues. One important difference is that biochars derived from livestock manure usually have high ash content^19^. Additionally, biochars based on livestock manure are richer in essential nutrients than plant-based biochars^26^. Biochar is increasingly received attention and is highly recommended as a soil amendment because it not only improves soil properties but also enhances soil fertility and crop productivity by improving nutrients retention^27,28^. Besides, using biochar as soil amendment can also release nutrients to provide a carbon and energy source for microbial growth^28,29^. Hence, biochar can be utilized as a fertilizer as well as a heavy metal stabilizer.

In a previous study, we obtained a mutant genotype (B38) from wild-type *Bacillus subtilis* using UV irradiation^10^. The mutant genotype exhibited a resistance to Cd greater than 4-fold that of the original wild-type species. In our previous study, we found that B38 has a high biosorption capacity for Cd, Cr, Hg, and Pb ions in solution^30^. The applicability of this mutant genotype to the immobilization of heavy metals assisted by biochar was investigated in the present study. Two types of biochars, one derived from plant residue and one from livestock manure, were used as carriers of B38 to enhance the bioaugmentation efficiency. The present study aims to develop a bioaugmentation technology that solves multiple heavy metal contamination in soil.

## Results and Discussion

### Promotion of plant growth in pot cultures

Six treatments were designed for the study: a control (S), soil amended only with B38 (SB), soil amended only with corn straw derived biochar (SC), soil amended with both B38 and corn straw-derived biochar (SCB), soil amended only with pig manure-derived biochar (SP), and soil amended with both B38 and pig manure-derived biochar (SPB).

**Figure 1:**
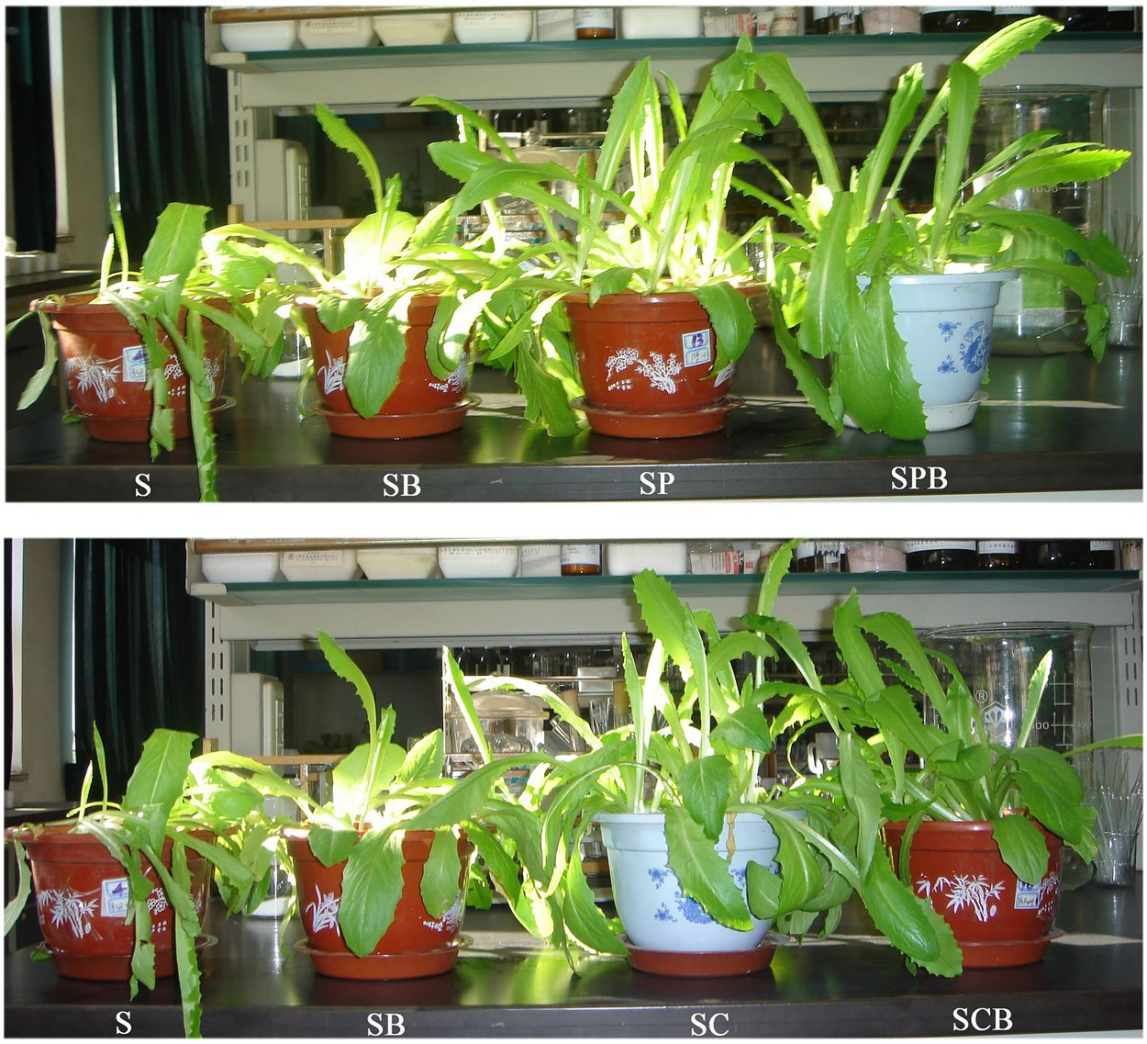
The photograph of lettuce grown in soils of different treatments. (S: control; SB: soil amended with B38; SC: soil amended with corn straw-derived biochar; SCB: soil amended with corn straw-derived biochar and B38; SP: soil amended with pig manure-derived biochar; SPB: soil amended with pig manure-derived biochar and B38).

**Figure 2:**
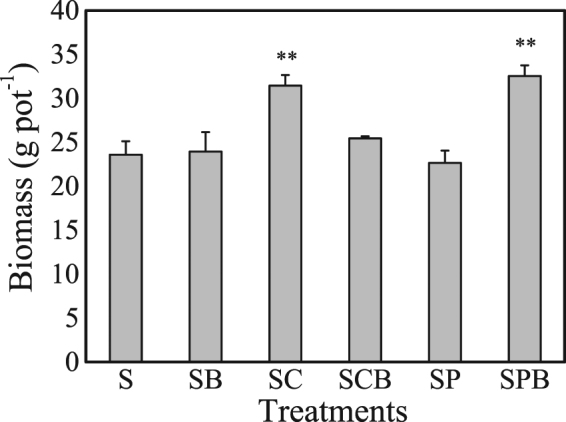
The edible part biomass of lettuce on dry weight basis (S: control; SB: soil amended with B38; SC: soil amended with corn straw-derived biochar; SCB: soil amended with corn straw-derived biochar and B38; SP: soil amended with pig manure-derived biochar; SPB: soil amended with pig manure-derived biochar and B38) (**= the probability level of *P* < 0.05) (*n* = 3).

The growth of lettuce was obviously promoted by B38 and biochar compared to the control (S) (Fig. 1). The biomass of the edible part of the lettuce grown under the different treatments is shown in Fig. 2. A significant increase in the plant biomass was observed when the soil was amended with the corn straw-derived biochar (SC), compared to the control (S) (*P* = 0.004). However, the plant biomass decreased compared to S under the treatment with pig manure-derived biochar (SP). The corn straw-derived biochar and pig manure-derived biochar was neutral (pH = 7.5) and alkalescent (pH = 8.1), respectively, and the CEC and OM content of corn straw-derived biochar were higher than in the pig manure-derived biochar (Table 1). Thus, corn straw-derived biochar demonstrated positive properties for increasing soil fertility to promote the growth of the plants. However, the edible biomass of the lettuce in SCB was higher than that in SB but lower than that in SC. Thus, corn straw-derived biochar used as a carrier could promote the activity of B38, but it reduces the improvement effect of soil fertility compared with the corn straw-derived biochar alone.

**Table 1:** Physico-chemical properties and heavy metal concentrations of soil and biochars.

		Soil	Corn straw-derived biochar	Pig manure-derived biochar
pH^a^		7.4	7.5	8.1
OM^b^ (%)		1.6	94.8	52.7
DOC (mg C L^−1^)		99.5	ND^c^	ND
Texture (%)				
Sand		10.6	ND^c^	ND
Silt		31/2	ND	ND
Clay		58.2	ND	ND
CEC^d^ (cmol kg^−1^)		17.5	108.0	64.0
Ash (%)		ND	13.6	39.4
S_BET_^e^ (m^2^ g^−1^)		ND	10.7	26.8
Mineral composition (g/kg)				
N		2.06	9.26	31.55
P		3.02	5.95	51.70
K		16.51	7.46	17.37
S		0.03	0.98	1.54
Ca		4.15	7.29	78.10
Mg		6.84	2.38	15.78
Zn		0.06	—	—
Cu		0.02	—	—
Mn		0.44	0.45	0.62
Fe		27.99	5.25	24.47
Heavy metal concentration (mg kg^−1^)				
Cd	Sample	2.1	4.6	3.7
	Limit^f^	0.4		
Cr	Sample	83.0	68.0	43.0
	Limit	200		
Hg	Sample	0.7	24.3	11.2
	Limit	0.4		
Pb	Sample	51.0	19.0	12.0
	Limit	50.0		

^a^pH, was measured using soil in 1:2.5 (w/v) 0.01 M CaCl_2_ solution. ^b^OM, organic matter. ^c^ND, not determined. ^d^CEC, cation exchange capacity. ^e^Surface area determined by N_2_ adsorption using the Brunauer-Emmett-Teller (BET) method. ^f^Limit established by Ministry of Environmental Protection of the People’s Republic of China for agricultural production and based on protection of human health (GB15618–2008).

There was no significant difference (*P* > 0.05) in the edible biomass of the lettuce in SB compared to that of S (Fig. 2). The nutrient element contents of the soil are shown in Table 1. The nutrient contents of the soil were slightly lower than the mean level of other farmland soils without fertilization^31–33^. Hence, when B38 was amended without a nutrient source, there were not sufficient nutrients to meet the needs of B38 growth. When amending the pig manure-derived biochar with B38, the biomass of the edible part increased obviously. The ash content of the pig manure-derived biochar was much higher than of the corn straw-derived biochar, and most of the mineral element contents of the pig manure-derived biochar were higher than those of the corn straw-derived biochar (Table 1). This suggests that there were abundant mineral components in the pig manure-derived biochar. Thus, pig manure derived biochar enhanced the B38 activity and increased the soil fertility. Lu *et al*.^1^ investigated the effects of biochars derived from bamboo and rice straw on the growth of *Sedum plumbizincicola* in a sandy loam paddy soil naturally contaminated with heavy metals. The treatment with rice straw biochar (loading rates at 5% w/w) produced the highest dry weight of shoot, which was 43% more than the control treatment. In this study, when pig manure-derived biochar combined with B38 was used as a co-amendment (with loading rates at 2% w/w), the growth of the biomass in the edible part of the lettuce reached 37.9% compared to that in S. Thus, pig manure derived-biochar is an excellent carrier for B38, and a synergistic effect in plant growth promotion was exhibited.

### Inhibition of heavy metal sorption

After the amendment with B38 and/or biochar, the heavy metal concentrations in the edible part of the lettuce decreased compared to the control (Table 2). However, the reductions in heavy metal uptake by the edible part of the lettuce after bacterial inoculation (SB) were less than other treatments and ranged from 15.16% to 49.37%. In a previous study, the FT-IR spectrum revealed that some functional groups existed on the cell wall of B38, including the hydroxyl, carbonyl, carboxyl, and primary amide groups, which enabled the interaction of B38 with the heavy metal ions to form complexes^30^. Thus, B38 immobilizes heavy metals in soil, reducing the uptake by plants.

**Table 2:** Heavy metal concentrations in the edible part of lettuce of different treatments (S: control; SB: soil amended with B38; SC: soil amended with corn straw-derived biochar; SCB: soil amended with corn straw-derived biochar and B38; SP: soil amended with pig manure-derived biochar; SPB: soil amended with pig manure-derived biochar and B38) (*n = *3).

heavy metal	Cd			Cr			Hg			Pb		
	concentration(mg kg^−1^)	reduction (%)	Limit^a^(mg kg^−1^)	concentration(mg kg^−1^)	reduction (%)	Limit^a^(mg kg^−1^)	concentration(mg kg^−1^)	reduction (%)	Limit^a^(mg kg^−1^)	concentration(mg kg^−1^)	reduction (%)	Limit ^a^(mg kg^−1^)
S	0.52 ± 0.03	—	0.2	4.42 ± 0.05	—	0.5	0.79 ± 0.02	—	0.01	5.33 ± 0.04	—	0.3
SB	0.40 ± 0.01	23.08		3.75 ± 0.07	15.16		0.40 ± 0.02	49.37		4.14 ± 0.22	22.33	
SC	0.32 ± 0.02	37.96		3.18 ± 0.11	27.82		0.59 ± 0.01	25.96		0.89 ± 0.03	83.26	
SCB	0.16 ± 0.01	68.64		2.62 ± 0.15	40.69		0.41 ± 0.03	48.94		0.33 ± 0.01	93.73	
SP	0.17 ± 0.04	67.39		2.25 ± 0.41	49.04		0.34 ± 0.01	57.68		0.27 ± 0.03	94.90	
SPB	0.06 ± 0.01	87.65		1.33 ± 0.01	69.95		0.15 ± 0.02	80.66		0.21 ± 0.01	96.06	

^a^Limit established by Ministry of Health of the People’s Republic of China for the maximum levels of contaminants in foods (GB2762–2012).

The heavy metal concentrations in the edible part of lettuce from the six treatments followed the order SPB < SP < SCB < SC < SB < S. There are some functional groups on the biochar surface, such as the hydroxyl, carbonyl, and carboxyl groups, which are involved in ion exchange, electrostatic, and cation-π interactions with heavy metals in soil^34,35^. Thus, biochar, as a carrier of B38, could enhance the activity of exotic microorganisms and increase the immobilization capacity of heavy metals. Moreover, among the six treatments, the SPB treatment showed the greatest inhibition on heavy metal accumulation by the plant. Generally, the micropore amount is related to the surface area for a porous material. The surface area of pig manure-derived biochar was approximately 2-fold higher than that of the corn straw-derived biochar (Table 1). Thus, there were more sorption sites in the pig manure-derived biochar surface to provide more adhesion space for B38 growth and reproduction. Mineral impurities (ash) served as the main biochar adsorption sites for the heavy metal ions adsorption^20^. The ash content of the pig manure-derived biochar was 3-fold higher than that of the corn straw-derived biochar. Thus, the immobilization capacity of heavy metals by the pig manure-derived biochar was much higher than that of the corn straw-derived biochar, and the pig manure-derived biochar provided more nutrients for B38 growth to utilize its activity. Bian *et al*.^36^ conducted a field experiment to confirm the immobilization of Cd and Pb in a contaminated paddy field with wheat straw biochar. Under the biochar treatments (loading rates at 10, 20, and 40 t ha^−1^), the total Cd and Pb uptakes by rice were reduced by 27–67% and 27–69%, respectively. In this study, when the pig manure-derived biochar combined with B38 was used as a co-amendment (with loading rates at approximate 10 t ha^−1^), the reduction of Cd and Pb concentrations in the edible part of the lettuce reached 87.7% and 96.1%, respectively, compared to that of the S. Moreover, after the pig manure-derived biochar treatment, the Cd and Pb concentrations in the edible part of the lettuce reached the standard levels of China (Table 2). Thus, the pig manure-derived biochar amended with B38 exhibited a synergistic effect on heavy metal immobilization in the soil.

### Reduction of heavy metal bioavailability

The amendments showed significant effects on the inhibition of heavy metal bioaccumulation by plants (*P* > 0.05). This indicates that the speciation and bioavailability of the heavy metals have changed in the soil. The extractable fractions of heavy metals were obtained by several methods to evaluate the speciation variations of heavy metals during bioremediation (Table 3). Mehlich 3 (M3) is a complex extractant and can extract exchangeable and organically bound fractions^37^. The high concentration (0.11 mmol L^−1^) of acetic acid in the first step of the extraction method developed by the Community Bureau of Reference (BCR1) enables it to partially extract the organic matter bound fraction^38^ and most metal fractions associated with calcium carbonate and minerals (kaolinite, potassium feldspar and ferrihydrite)^39^. DTPA is a chelating agent and can release the soluble, exchangeable, adsorbed and organically bound fractions, as well as some of the fractions fixed by metal oxides^40^.

**Table 3:** Results of extractable heavy metals of rhizosphere soils, and the linear correlation coefficients (*r* ^2^) between metal concentrations in the edible part of the lettuces and extracted metals from soils by different extraction methods (S: control; SB: soil amended with B38; SC: soil amended with corn straw-derived biochar; SCB: soil amended with corn straw-derived biochar and B38; SP: soil amended with pig manure-derived biochar; SPB: soil amended with pig manure-derived biochar and B38) (*n* *=* 3).

Treatment		Extracted metal concentration (mg/kg)						*r* ^2^
		S	SB	SC	SCB	SP	SPB	
DTPA	Cd	0.88 ± 0.22	0.55 ± 0.06	0.48 ± 0.02	0.32 ± 0.05	0.45 ± 0.05	0.31 ± 0.08	0.84*
	Cr	0.62 ± 0.07	0.38 ± 0.02	0.37 ± 0.02	0.33 ± 0.06	0.34 ± 0.04	0.38 ± 0.02	0.45
	Hg	0.07 ± 0.02	0.04 ± 0.01	0.05 ± 0.01	0.04 ± 0.02	0.04 ± 0.02	0.03 ± 0.01	0.94**
	Pb	3.00 ± 0.13	2.61 ± 0.12	2.46 ± 0.17	2.17 ± 0.10	2.24 ± 0.13	2.00 ± 0.14	0.85*
M3	Cd	1.65 ± 0.08	1.38 ± 0.08	0.50 ± 0.09	0.48 ± 0.02	0.57 ± 0.03	0.40 ± 0.04	0.80
	Cr	6.10 ± 0.42	5.54 ± 0.56	5.70 ± 0.23	3.19 ± 0.21	5.32 ± 0.25	4.84 ± 0.13	0.25
	Hg	0.08 ± 0.01	0.08 ± 0.02	0.07 ± 0.01	0.05 ± 0.03	0.07 ± 0.02	0.04 ± 0.02	0.49
	Pb	2.41 ± 0.43	1.64 ± 0.32	1.01 ± 0.08	0.93 ± 0.08	1.03 ± 0.06	0.89 ± 0.03	0.93**
BCR1	Cd	1.53 ± 0.03	1.30 ± 0.12	1.38 ± 0.11	1.17 ± 0.08	1.37 ± 0.05	1.19 ± 0.04	0.63
	Cr	6.48 ± 0.07	6.18 ± 0.34	5.89 ± 0.18	5.14 ± 0.19	5.51 ± 0.26	4.71 ± 0.79	0.91**
	Hg	0.07 ± 0.02	0.07 ± 0.01	0.06 ± 0.02	0.06 ± 0.01	0.06 ± 0.02	0.05 ± 0.02	0.51
	Pb	4.15 ± 0.11	2.75 ± 0.09	2.76 ± 0.03	1.19 ± 0.03	1.18 ± 0.22	0.99 ± 0.02	0.78

* and ** represent the probability level of *P* < 0.1 and *P* *<* 0.05, respectively.

Generally, M3 and BCR1 can extract more extractable Cd and Cr than can DTPA. The extractable contents of Cd and Cr versus total contents in the soil were 70% and 7%, respectively (Table 3). The extractable Hg and Pb contents extracted by the three methods was similar (Table 3). The extractable contents of Hg and Pb were approximately 5% and 10%, respectively. The bioavailable contents of heavy metals all decreased after treatments by both single amendments and the 2 co-amendments, and followed the order SPB < SCB < SP < SC. After remediation, the risk of heavy metals to the environment and human was reduced. Moreover, the heavy metal remediation efficiency of the biochar was enhanced after combining it with B38. Jiang *et al*.^41^ developed rice straw-derived biochar for the remediation of Cu, Cd and Pb polluted soil. When 3% and 5% biochar were added, the acid soluble Cd and Pb (extracted by 0.11 mmol/L acetic acid) decreased by 5.6–14.1% and 18.8–77.0%, respectively. In this study, when 2% biochar was added alone, the bioavailable contents of Cd and Pb were decreased by 9.8–10.5% and 33.7–71.7%, respectively. After amending with 2% co-amendments, the reduction of bioavailable contents of Cd and Pb reached 22.2–23.5% and 71.3–76.1%, respectively. Thus, the co-amendments reduced heavy metal bioavailability efficiently.

The use of chemical extraction to predict the bioavailability of heavy metals to plants has long been disputed because the amounts extractable by chemical extraction methods often do not show significant correlation with those bioaccumulated in plants. A universal method has not yet been accepted, and might not be achieved in future. The correlations between the extractable metals and metal concentrations in the edible part of the lettuce were analyzed for the six different treatments (Table 3). Generally, it was demonstrated that DTPA was a good extractant for predicting the heavy metal cation bioavailability of the leafy vegetable, and BCR1 was a good extractant for predicting the heavy metal anion bioavailability of the leafy vegetable. Moreover, M3 showed excellent capacity for predicting the Pb bioavailability.

### DGGE analyses of B38 addition to soil microorganisms

**Figure 3:**
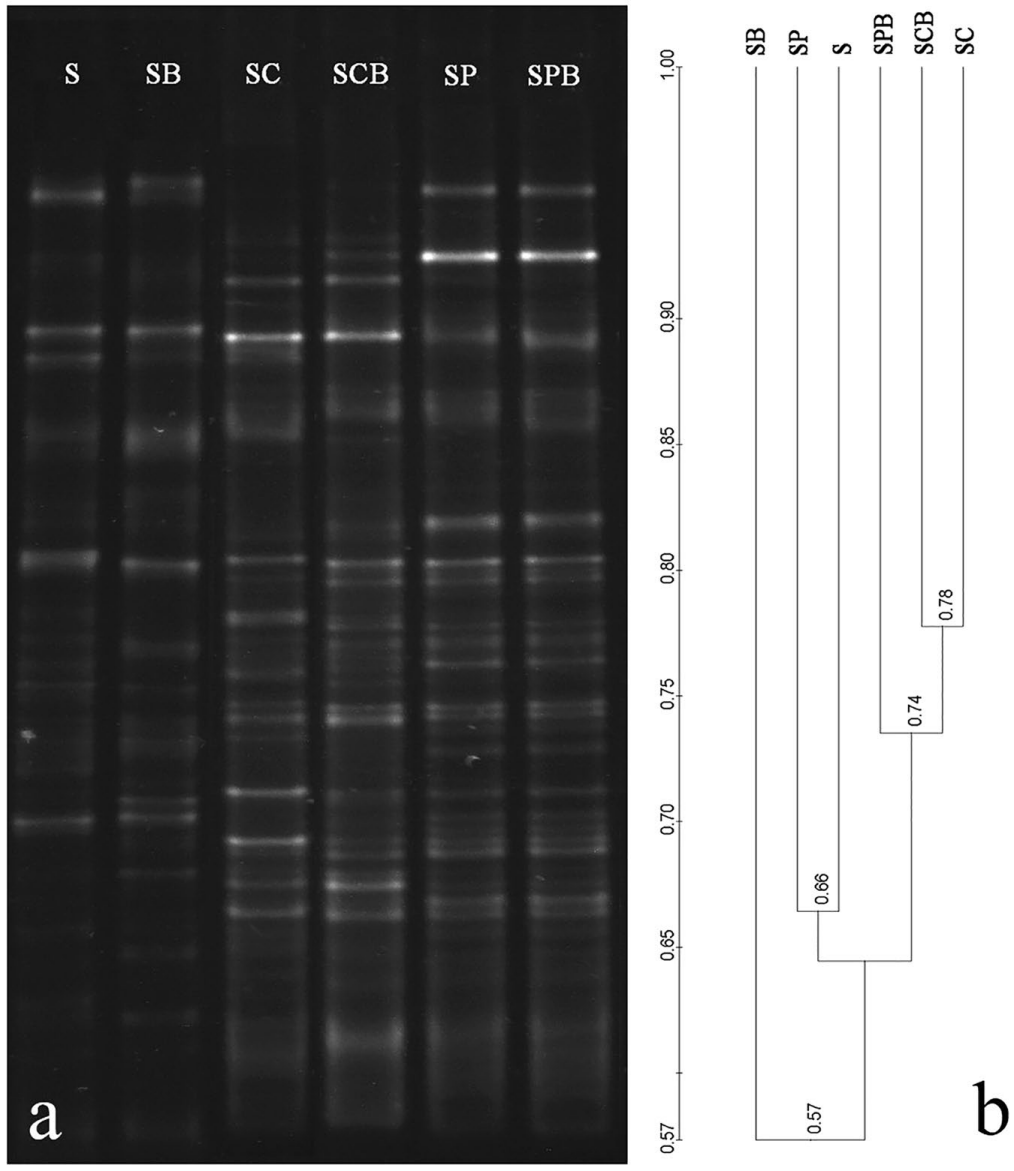
DGGE separation patterns of PCR amplified 16S rDNA fragments from rhizosphere soil samples (**a**) and cluser analysis diagram of DGGE profiles (**b**) with different treatments (S: control; SB: soil amended with B38; SC: soil amended with corn straw-derived biochar; SCB: soil amended with corn straw-derived biochar and B38; SP: soil amended with pig manure-derived biochar; SPB: soil amended with pig manure-derived biochar and B38).

To further clarify the mechanism of heavy metal immobilization by the amendments, and to elucidate the possible effect of the amendments on the soil microbial community, the microbial community compositions under the different treatments were analyzed by PCR-DGGE. The bacterial DGGE profiles revealed the structural composition of the communities in the soil samples. Each of the distinguishable bands in the separation pattern represents an individual bacterial species. The relative intensities of the bands among the soil samples under the six different treatments were quite different, indicating the differences in their bacterial densities (Fig. 3a).

The bands in the DGGE profile of the amplified 16S rDNA gene fragments of the control soil samples (S) were few and weak, suggesting that the bacterial functional diversity was quite low in heavy metal-contaminated soils. When B38 was added alone (SB), the DGGE profile increased slightly compared to the control (S). This implied that the exotic microorganisms, B38, did not obviously enhance the soil microbial diversity. When the biochar was added alone (SC or SP), the DGGE profile increased obviously compared to that of the control (S), suggesting that biochar provides nutrients or immobilizes heavy metals, which are beneficial for the growth of native microorganisms. When B38 and biochar were co-applied (SCB or SPB), the DGGE profile was more abundant and more intense than in the other four treatments. Hence, it can be concluded that the combined technology of SCB and SPB substantially inhibited the adverse effect of heavy metal contamination on soil microbes and enhance the proliferation of soil microbes. The microbial community results under the different treatments are in accordance with the improvement efficiencies of plant growth and the immobilization efficiencies of heavy metals. Moreover, the treatment with the greatest microbial activities (SPB) showed the least heavy metal adsorption and the greatest plant growth, demonstrating again that SPB is a good additive for soils contaminated by heavy metals.

**Figure 4:**
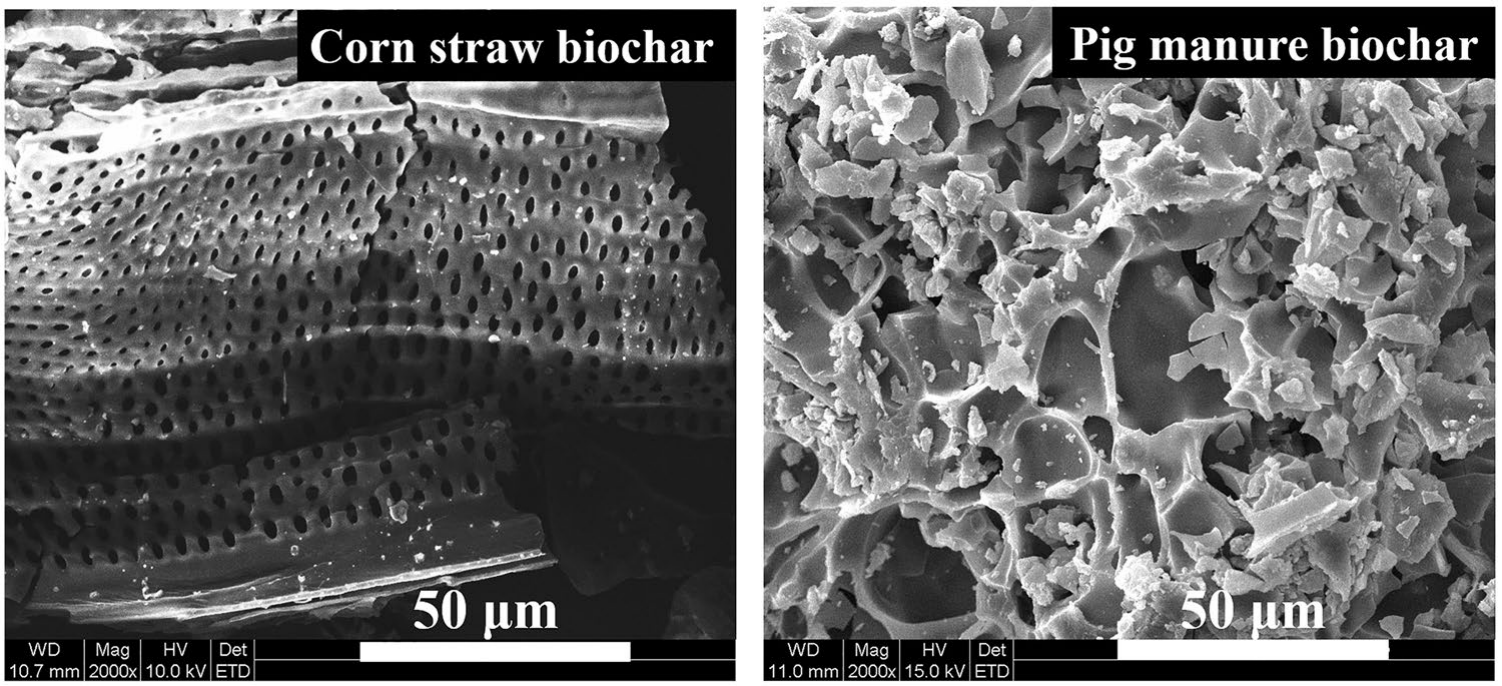
SEM photography of biochar samples derived from different stock materials.

The cluster analysis of the intensity of the DGGE bands is shown in Fig. 3b. The bacterial communities formed two major clusters: S, SB and SP; SC, SCB and SPB. In nutrient deficient soils (S and SB), neither exotic B38 nor native microbes grew well. With the SC and SCB treatments, corn straw-derived biochar provided sufficient nutrients for the growth of both the exotic B38 and native microbes. However, when the pig manure-derived biochar was used as an external nutrient source and added alone (SP), the microbial diversity did not increase significantly. However, with the SPB treatment, the microbial diversity increased obviously. Thus, pig manure-derived biochar was a good carrier for B38, and the surface characteristic of pig manure-derived biochar might change after combining it with B38. The SEM photographs showed obvious differences in the structures of the corn straw-derived biochar and the pig manure derived biochar (Fig. 4). The corn straw-derived biochar materials looked like the sieve plates with a polyporous structure, while the pig manure-derived biochar materials were a polyporus structure with a lacunose surface. Thus, the B38 mutant not only covers on the surface of corn straw-derived biochar and pig manure-derived biochar but also partially inhabits the micropores in the pig manure-derived biochar. This could relieve the competition with the native microbes.

The molecular analysis validates that microbial diversity increased and the microbial communities changed after bioremediation. Combined with the above inhibition of heavy metal bioaccumulation in plants, this result illustrates that special metal-resistant bacteria (B38 in this study) is a promising approach for ecological remediation. The co-amendment of pig manure-derived biochar and B38 could alleviate the effects of heavy metals in soils, which is a beneficial microorganism propagation-promoting amendment.

## Conclusions

A mutant species from *B*. *subtilis* (B38) acquired by UV irradiation was confirmed to be a suitable adsorbent for both cationic and anionic heavy metals (Cd, Hg, Pb, and Cr). Two types of biochars, one derived from corn straw and one from pig manure, were used as the carriers of B38. After the amendment of the biochar, the bioavailability of heavy metal contaminations decreased. Moreover, the heavy metal remediation efficiency of the biochar was enhanced after combining it with B38. The simultaneous application of B38 and pig manure-derived biochar exhibited a superior effect on the immobilization of heavy metals in soil. The plant biomass with the pig manure-derived biochar treatment increased by 37.9% compared to the control and the heavy metal concentrations in the edible part of the lettuce decreased by 69.95–96.06%. The PCR-DGGE profiles revealed that the pig manure-derived biochar enhanced the proliferation of both the exotic B38 and native microbes. From these results, the pig manure-derived biochar a good carrier for the bacterium. The pig manure-derived biochar amended with B38 simultaneously exhibited a synergistic effect in the soil fertility improvement, plant growth promotion, and soil heavy metal immobilization. These results suggest that B38 carried by the pig manure-derived biochar may be a promising candidate for the remediation of soils contaminated by multiple heavy metals.

## Methods

### Microorganism, culture condition and preparation of biosorbent

The bacterium used in this study was a mutant genotype (B38) of *Bacillus subtilis* obtained by UV irradiation. The wild type of *B*. *subtilis* strain was purchased from China General Microbiological Culture Collection Center (CCGMC). Briefly, *B*. *subtilis* was irradiated by shortwave UV light (254 nm) at room temperature in the dark under mild agitation for 1–10 min, and then conducting two rounds screening by incubating the mutant strains in spiked culture medium with 0.3 mmol L^−1^ Cd to acquire the high tolerant and stable strains^10^. This mutant genotype exhibits high resistance to Cd and can tolerate 3 mmol L^−1^ Cd in solution, while the original wild type of *B*. *subtilis* can only tolerate 0.25 mmol L^−1^ Cd. In a previous study, we examined the maximum tolerant concentration of B38 to other metals, and the values were found to be 4 mmol L^−1^ Cr, 0.5 mmol L^−1^ Hg, and 4.5 mmol L^−1^ Pb in solution^42^. Moreover, the B38 has high adsorption capacities for Cd, Cr, Hg, and Pb ions in solution^30^. The B38 cells were cultured for 24 h in a beef extract-peptone broth medium (peptone, 10 g L^−1^; beef extract, 5 g L^−1^; and NaCl, 10 g L^−1^) in a shaker operated at 37 °C and 200 rpm to the late exponential phase.

### Biochar preparation

Corn straw stock was collected from a farmland in Jinnan District, Tianjin, China. Pig manure stock was collected from a hoggery in Jixian County, Tianjin, China. The corn straw and pig manure were air-dried and ground to pass through a 2 mm sieve, then heated at 350 °C in a ceramic pot covered with a tight-fitting lid (where oxygen was soon exhausted) in a preheated muffle furnace for 2 h^43^. The biochar produced were ground to pass through the 200–400 mesh sieves and stored in amber glass bottles.

### Characterization of soil and biochar

The soil used was collected from an agricultural plot in Jinnan District, Tianjin, China. Due to the sewage irrigation using water from the Dagu drainage canal, the soil was severely contaminated by Cd, Cr, Hg, and Pb, with concentrations of 2.1 mg kg^−1^, 83.0 mg kg^−1^, 0.7 mg kg^−1^ and 51.0 mg kg^−1^, respectively (Table 1). The concentrations of Cd, Hg, and Pb exceeded the level II soil standard (soil for agricultural activity with pH value 6.5–7.5) in China (GB15618–2008). The soil used in this study was classified as a clay according to the soil texture triangle and was classified as Alfisols according to the USDA Soil Taxonomy system. The soil was air dried and ground to pass through a 2 mm sieve. The physico-chemical properties of the soil and biochar, and the heavy metal concentrations in the soil are listed in Table 1. The surface morphology of the biochars was investigated by scanning electron microscopy (SEM, QUANTA 200, United States).

### Soil treatments

**Figure 5:**
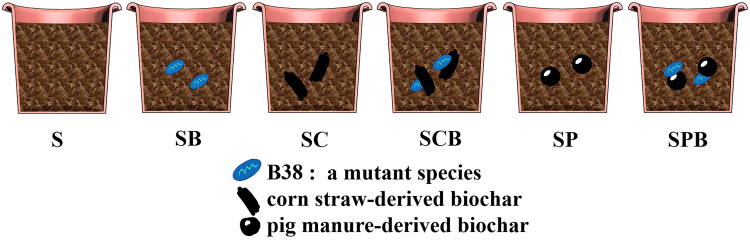
Design of the experiment.

Six treatments were designed for the study: a control (S), soil amended only with B38 (SB), soil amended only with corn straw-derived biochar (SC), soil amended with both B38 and corn straw-derived biochar (SCB), soil amended only with pig manure-derived biochar (SP), and soil amended with both B38 and pig manure-derived biochar (SPB) (Fig. 5). Eight hundred grams of soil sample was placed into a 1 L beaker. Then, 20 g kg^−1^ corn straw derived-biochar or pig manure-derived biochar and 20 mL kg^−1^cultural suspension (5 × 10^8^ cells mL^−1^) of B38 were amended according to the design. The B38 cultural suspension was mixed with corn straw-derived biochar or pig manure-derived biochar first, and then the mixture was added into the soil samples. The soil samples were mixed well with a glass rod and incubated in a growth chamber (LRH-800-GS, Guangdong Medical Instrument Company, China) at 25 ± 1 °C with a light cycle of 12:12 for 2 weeks to allow the amendments to mix well with the soil. Every 2 days, the beakers were supplemented with a specified amount of water to make up the loss of water.

### Extraction of bioavailable metals in soil

Two one-step extraction methods using DTPA and Mehlich 3 (M3) as extractants and the first step of the extraction method developed by the Community Bureau of Reference (BCR1) were compared for the evaluation of metal bioavailability in the soil.

The extraction by DTPA was conducted as following procedure: 30 mL of DTPA solution (0.005 mol L^−1^ DTPA + 0.1 mmol L^−1^ triethanolamine + 0.01 mmol L^−1^ CaCl_2_; buffered to pH 7.3 with dilute HCl) was added into 15 g of the treated soil sample, and shaken end-over-end for 2 h (200 rpm, 25 °C)^10^. The sample was centrifuged and the supernatant was analyzed for Cd, Cr, Hg and Pb concentrations.

The BCR step 1 (acid extractable/exchangeable fraction) was performed by adding 40 mL of 0.11 mmol L^−1^ acetic acid to 1 g of soil sample in a centrifuge tube and shaking for 16 h at room temperature. The extract was then separated from the solid residue by centrifugation (3000 rpm, 20 min) and the supernatant liquid was analyzed^35,44^.

The M3 extracting solution was composed of 0.2 mmol L^−1^ CH_3_COOH, 0.25 mmol L^−1^ NH_4_NO_3_, 0.015 mmol L^−1^ NH_4_F, 0.013 mmol L^−1^ HNO_3_ and 0.001 mmol L^−1^ ethylene diamine tetraacetic acid (EDTA). The extraction was conducted as follows: 20 mL of M3 solution was added into 2 g of air-dried soil sample, and shaken end-over-end for 3 min (200 rpm, 25 °C). The sample was centrifuged and the supernatant was analyzed^45^.

### Pot experiment

The pot experiment was carried out on a soil polluted by four heavy metals (Cd, Cr, Hg and Pb). A type of leafy vegetable, lettuce (*Lactuca sativa* L.), was studied in the pot experiment. The pots have drainage holes to ensure the health of plants. The pot experiment was conducted in a growth chamber (LRH-800-GS, Guangdong Medical Instrument Company, China) at 25 ± 1^◦ ^C with a light cycle of 12:12. Every 2 days, the pots were supplemented with a specified amount of deionized water to keep the relative moisture of the soils at approximately 60% of water holding capacity. The design of the experiment employed three replicates for each treatment in a completely randomized block design. The seeds were previously sterilized as described by Khan *et al*.^46^. Seedlings were sown in each pot with the treated soils on March 1, 2013, and the plants were thinned to five uniform seedlings per pot after 2 weeks. Eight weeks after planting (May 10), the plants were harvested. The plants were first washed thoroughly with tap water, then with deionized water, and were then freeze-dried. The rhizosphere soil was obtained by gently shaking off the loosely bound soil, while the rhizosphere soil adhering to the root was isolated by more vigorous shaking or by scraping. Moist rhizosphere soil was placed in a polyethylene bag and stored at −18 °C before analysis.

### Basic analysis for samples

The soil and plant sample was digested with a microwave digestion system (Yiyao WX4000, Shanghai, China). Details of the digestion methods were as previously described by Wang *et al*.^42^. All the digested samples were filtered using a 0.45 μm cellulose-acetate membrane. The concentrations of Cd, Cr and Pb were determined using an atomic absorption spectrophotometer (AAS, Rayleigh WFX 210, Beijing, China). The concentration of Hg was determined using an atomic fluorescence spectrometer (AFS, Jitian AFS 830, Beijing, China).

### Soil DNA extraction, purification, quantification and denaturing gradient gel electrophoresis (DGGE) analysis

The soil microbial community compositions were analyzed using PCR-based polymerase chain reaction-denaturing gradient gel electrophoresis (PCR–DGGE) to assess the potential effect of remediation on the soil microorganism community.

The total community DNA from the soil was extracted using Omega DNA Extraction Kit (Omega Biotek, Doraville, GA, USA) following the manufacturer’s instruction.

Prime F338 was used in this study for the amplification of bacterial 16S rDNA gene. The variable V3 region of the domain bacteria was amplified using PRBA338F (5′-CCT ACG GGA GGC AGC AG-3′) and PRUN518R (5′-ATT ACC GCG GCT GG-3′) primers with a GC clamp attached to the forward primer^47^. PCR reaction was executed in a PTC thermal cycler (Bio-Rad Laboratories, Hercules, CA, USA) in a 0.2 mL tube using a 50 μL reaction volume. The reaction mixture contained 25 pmol of both the primers, 20 μmol of each dNTPs, 5 μL of 10 × reaction buffer, and 2.5 units of Taq DNA polymerase. The cycling conditions used to amplify the 16S rDNA gene fragment were 94 °C for 5 min, followed by 35 cycles of 94 °C for 1 min, 55 °C for 1 min and 72 °C for 2 min. A final extension period of 72 °C for 10 min was used. An aliquot of 5 μL PCR products was checked by electrophoresis in 1.5% (w/v) agarose gels stained with ethidium bromide prior to DGGE analysis^48^.

For the DGGE analysis, the PCR product generated from each sample was separated on a 8% acrylamide gel (acrylamide: bisacrylamide, 37.5:1) with a linear denaturant gradient range from 30% to 60% using 30 μL of the PCR product in a 1 × TAE buffer at 60 °C, and 160 V for 300 min. Gels were stained with ethidium bromide stain and the gels were scanned (Bio-Rad Laboratories, Hercules, CA, USA).

Digitized DGGE images were analyzed with Quantity One image analysis software (version 4.0, Bio-Rad Laboratories, Hercules, CA, USA).

### Statistical analysis

Statistical significances were determined using one-way analysis of variance (ANOVA) followed by Tukey’s test for multiple comparisons, with a *P* value < 0.05, using OriginPro 8.5 software.

## References

[1] Lu K (2014). Effect of bamboo and rice straw biochars on the bioavailability of Cd, Cu, Pb and Zn to *Sedum plumbizincicola*. Agr. Ecosyst. Environ..

[2] Ledin M, Krantz-Rülcker C, Allard B (1999). Microorganisms as metal sorbents: comparison with other soil constituents in multi-compartment systems. Soil. Biol. Biochem..

[3] Udeigwe TK (2011). Application, chemistry, and environmental implications of contaminant-immobilization amendments on agricultural soil and water quality. Environ. Int..

[4] Akcil A (2004). Potential bioleaching developments towards commercial reality: Turkish metal mining’s future. Miner. Eng..

[5] El-Bestawy E (1998). Enhancement of bacterial efficiency for metal removal using mutation techniques. World. J. Microb. Biot..

[6] Valls M, De Lorenzo V (2002). Exploiting the genetic and biochemical capacities of bacteria for the remediation of heavy metal pollution. FEMS. Microbiol. Rev..

[7] Brierley JA (2008). A perspective on developments in biohydrometallurgy. Hydrometallurgy..

[8] Qiu GZ, Liu XD, Zhou HB (2008). Microbial community structure and function in sulfide ore bioleaching systems. T. Nonferr. Metal. Soc..

[9] Yang YN (2007). Mutation effect of MeV protons on bioflocculant bacteria *Bacillus cereus*. Nucl. Instrum. Meth. B..

[10] Jiang C (2009). Immobilization of cadmium in soils by UV-mutated *Bacillus subtilis* 38 bioaugmentation and NovoGro amendment. J. Hazard. Mater..

[11] Yin H (2008). Improvement of chromium biosorption by UV–HNO_2_ cooperative mutagenesis in Candida utilis. Water. Res..

[12] Reddy, K. & Cutright, T. Nutrient amendment for the bioremediation of a chromium-contaminated soil by electrokinetics. *Energ*. *Source*. **25**, 931-943 (2003).

[13] Roane TM, Josephson KL, Pepper IL (2001). Dual-bioaugmentation strategy to enhance remediation of cocontaminated soil. Appl. Environ. Micro..

[14] Pal S (2010). Use of bio-resources for remediation of soil pollution. Nat. Resource..

[15] Juwarkar AA, Jambhulkar HP (2008). Restoration of fly ash dump through biological interventions. Environ. Monit. Assess..

[16] Trigo C (2014). Influence of soil biochar aging on sorption of the herbicides mcpa, nicosulfuron, terbuthylazine, indaziflam, and fluoroethyldiaminotriazine. J. Agr. Food. Chem..

[17] Cao X (2011). Simultaneous immobilization of lead and atrazine in contaminated soils using dairy-manure biochar. Environ. Sci. Technol..

[18] Moreno-Jiménez E (2016). Availability and transfer to grain of As, Cd, Cu, Ni, Pb and Zn in a barley agri-system: impact of biochar, organic and mineral fertilizers. Agr. Ecosyst. Environ..

[19] Uchimiya M, Bannon DI, Wartelle LH (2012). Lead retention by broiler litter biochars in small arms range soil: impact of pyrolysis temperature. J. Agr. Food. Chem..

[20] Uchimiya M (2010). Immobilization of heavy metal ions (Cu-II, Cd-II, Ni-II, and Pb-II) by broiler litter-derived biochars in water and soil. J. Agr. Food. Chem..

[21] Han Y (2013). Heavy metal and phenol adsorptive properties of biochars from pyrolyzed switchgrass and woody biomass in correlation with surface properties. J. Environ. Manage..

[22] Lucchini P (2014). Does biochar application alter heavy metal dynamics in agricultural soil?. Agr. Ecosyst. Environ..

[23] Rombolà AG (2015). Relationships between chemical characteristics and phytotoxicity of biochar from poultry litter pyrolysis. J. Agr. Food. Chem..

[24] Brewer CE (2011). Criteria to select biochars for field studies based on biochar chemical properties. Bioenerg. Res..

[25] Spokas KA (2011). Qualitative analysis of volatile organic compounds on biochar. Chemosphere..

[26] Sarkhot DV, Berhe AA, Ghezzehei TA (2012). Impact of biochar enriched with dairy manure effluent on carbon and nitrogen dynamics. J. Environ. Qual..

[27] Lehmann J, Gaunt J, Rondon M (2006). Bio-char sequestration in terrestrial ecosystems - A review. Mitig. Adapt. Strat. Gl..

[28] Lehmann J (2011). Biochar effects on soil biota – A review. Soil. Biol. Biochem..

[29] Amit KJ (2017). Linking the belowground microbial composition, diversity and activity to soilborne disease suppression and growth promotion of tomato amended with biochar. Sci. Rep..

[30] Wang T, Sun HW (2013). Biosorption of heavy metals from aqueous solution by UV-mutant *Bacillus subtilis*. Environ. Sci. Pollut. R.

[31] Sager M (2007). The effect of soil bacteria and perlite on plant growth and soil properties in metal contaminated samples. Water. Air. Soil. Poll..

[32] Cavani L (2016). Ecological restoration of a copper polluted vineyard: long-term impact of farmland abandonment on soil bio-chemical properties and microbial communities. J Environ. Manage..

[33] Bedada W (2016). Soil nutrient build-up, input interaction effects and plot level n and p balances under long-term addition of compost and np fertilizer. Agr. Ecosyst. Environ..

[34] Cao XD (2009). Dairy-manure derived biochar effectively sorbs lead and atrazine. Environ. Sci. Technol..

[35] Xu XY (2016). Comparison of the characteristics and mechanisms of Hg(II) sorption by biochars and activated carbon. J. Colloid. Interf. Sci..

[36] Bian R (2014). A three-year experiment confirms continuous immobilization of cadmium and lead in contaminated paddy field with biochar amendment. J. Hazard. Mater..

[37] Bei W (1997). Chemical forms of cobalt and nickel extracted by M1, M3 and CaCl_2_-DTPA. J. Environ. Sci.-China..

[38] Payá-pérez A, Sala J, Mousty F (1993). Comparison of ICP-AES and ICP-MS for the analysis of trace elements in soil extracts. Int. J. Environ. An. Chem..

[39] Whalley C, Grant A (1994). Assessment of the phase selectivity of the European Community Bureau of Reference (BCR) sequential extraction procedure for metals in sediment. Anal. Chim. Acta..

[40] Lindsay WL, Norvell WA (1969). Development of a DTPA micronutrient soil test. Agron. Abstr..

[41] Jiang J (2012). Immobilization of Cu(II), Pb(II) and Cd(II) by the addition of rice straw derived biochar to a simulated polluted ultisol. J. Hazard. Mater..

[42] Wang T (2014). The immobilization of heavy metals in soil by bioaugmentation of a UV-mutant *Bacillus subtilis* 38 assisted by NovoGro biostimulation and changes of soil microbial community. J. Hazard. Mater..

[43] Keiluweit M (2010). Dynamic molecular structure of plant biomass-derived black carbon (biochar). Environ. Sci. Technol..

[44] Nemati K (2011). Speciation of heavy metals by modified BCR sequential extraction procedure in different depths of sediments from Sungai Buloh, Selangor, Malaysia. J. Hazard. Mater..

[45] Mehlich A (1984). Mehlich 3 soil test extractant: A modification of Mehlich 2 extractant. Commun. Soil. Sci. Plan..

[46] Khan S (2008). Accumulation of polycyclic aromatic hydrocarbons and heavy metals in lettuce grown in the soils contaminated with long-term wastewater irrigation. J. Hazard. Mater..

[47] Muyzer G, De Waal EC, Uitterlinden AG (1993). Profiling of complex microbial populations by denaturing gradient gel electrophoresis analysis of polymerase chain reaction-amplified genes coding for 16S rRNA. Appl. Environ. Micro..

[48] Chen YX (2006). Impacts of chelate-assisted phytoremediation on microbial community composition in the rhizosphere of a copper accumulator and non-accumulator. Sci. Total. Environ..

